# *Cryptosporidium* and *Giardia* in Humans, Domestic Animals, and Village Water Sources in Rural India

**DOI:** 10.4269/ajtmh.15-0111

**Published:** 2015-09-02

**Authors:** Miles E. Daniels, Arpit Shrivastava, Woutrina A. Smith, Priyadarshi Sahu, Mitsunori Odagiri, Pravas R. Misra, Pinaki Panigrahi, Mrutyunjay Suar, Thomas Clasen, Marion W. Jenkins

**Affiliations:** Department of Veterinary Medicine and Epidemiology, School of Veterinary Medicine, University of California Davis, Davis, California; School of Biotechnology, Kalinga Institute of Industrial Technology (KIIT), Odisha, India; Department of Civil and Environmental Engineering, University of California Davis, Davis, California; Asian Institute of Public Health, Bhubaneswar, Odisha, India; Departments of Epidemiology and Pediatrics, Center for Global Health and Development, College of Public Health, University of Nebraska Medical Center, Omaha, Nebraska; Department of Environmental Health, Rollins School of Public Health, Emory University, Atlanta, Georgia; Faculty of Infectious and Tropical Diseases, London School of Hygiene and Tropical Medicine, London, United Kingdom

## Abstract

*Cryptosporidium parvum* and *Giardia lamblia* are zoonotic enteric protozoa of significant health concern where sanitation, hygiene, and water supplies are inadequate. We examined 85 stool samples from diarrhea patients, 111 pooled fecal samples by species across seven domestic animal types, and water from tube wells (*N* = 207) and ponds (*N* = 94) across 60 villages in coastal Odisha, India, for *Cryptosporidium* oocysts and *Giardia* cysts to measure occurrence, concentration/shedding, and environmental loading rates. Oocysts/cysts were detected in 12% of diarrhea patients. Detection ranged from 0% to 35% for *Cryptosporidium* and 0% to 67% for *Giardia* across animal hosts. Animal loading estimates indicate the greatest contributors of environmental oocysts/cysts in the study region are cattle. Ponds were contaminated with both protozoa (oocysts: 37%, cysts: 74%), as were tube wells (oocysts: 10%, cysts: 14%). Future research should address the public health concern highlighted from these findings and investigate the role of domestic animals in diarrheal disease transmission in this and similar settings.

Diarrheal disease is a leading cause of mortality and morbidity worldwide for children under 5 years of age.^1^ Some of the most commonly detected pathogens associated with diarrhea in humans are the zoonotic protozoans *Cryptosporidium parvum* and *Giardia lamblia*.^2^,^3^ Poor hygiene and sanitation conditions and exposure to animals are factors thought to affect parasite transmission.^4^,^5^ These factors are relevant in the subcontinent of India, where the practice of open defecation and dispersed domestic animal feces creates a situation where exposure to *Cryptosporidium* and *Giardia* can be high.^6^

In conjunction with a large-scale trial of impacts of household sanitation on diarrheal disease in the Indian state of Odisha, in rural Puri District,^7^ we measured *Cryptosporidium* and *Giardia* in humans, domestic animals, and in surface and groundwater sources from villages in and around Puri District to assess the relevance of these pathogens for local disease burdens and the potential for zoonotic transmission from animals in study villages. The Ethical Review Committees of the Asian Institute of Public Health (AIPH) and London School of Hygiene and Tropical Medicine approved the study design and procedures.

Fresh stool samples from 85 diarrhea patients were collected at three local diarrhea wards from May to July 2012, with information on patient age (range: 6 months–79 years), gender (64% male), and residence (∼87% rural). Samples were placed on ice, transported to the AIPH laboratory within 4 hours of collection, and stored at 4°C up to 1 week until analyzed at the School of Biotechnology, Kalinga Institute of Industrial Technology (KIIT). Immunomagnetic separation (IMS) and direct immunofluorescence antibody tests (DFA) were used at KIIT to isolate, detect, and enumerate *Cryptosporidium* and *Giardia* oocysts/cysts in 5 g sample using methods previously reported.^8^ The frequency of *Cryptosporidium* and *Giardia* detection in diarrhea patients was each 12% with oocysts detected at higher concentrations compared with cysts (Table 1). Although multivariable logistic regression of demographic factors (age < 2 versus ≥ 2 years, gender, and rural versus urban location) for infection revealed no statistically significant results (*P* > 0.05), age < 2 (*N* = 30) had a marginally increased odds of *Cryptosporidium* infection (95% confidence interval [CI]: 0.8–17, *P* = 0.09) compared with age ≥ 2 (*N* = 55). This is consistent with The Global Enteric Multicenter Study (GEMS) identifying *Cryptosporidium* as the second largest cause of severe-to-moderate diarrhea in children < 2 years in India.^3^

Fresh fecal samples from 587 apparently healthy individual animals of seven domestic species were collected from dispersed sites across the study region and transported on ice and processed at KIIT similar to human fecal samples (above), after combining 4–10 individual samples from the same species (except cat) to create 111 pooled samples by species (see Table 1). To minimize risk of environmental contamination, freshly excreted fecal material was carefully collected from fecal surfaces that had not contacted the ground. Detection frequency and concentrations of *Cryptosporidium* and *Giardia* oocysts/cysts varied considerably between host species (Table 1). Using cattle (most commonly owned and abundant livestock in the region)^9^ as the referent in univariable logistic regression, sheep and goats had significantly greater odds of shedding *Cryptosporidium* (95% CI: 2.4–308.1) while dogs had significantly greater odds of shedding *Giardia* (95% CI: 1.6–21.0). The single pooled cat sample was excluded. Reports of *Cryptosporidium* among sheep and goats in India are lacking, and the zoonotic potential of *Cryptosporidium* in these animals is still unclear.^10^ Goats and sheep are most often recognized to shed host-specific *Cryptosporidium* genotypes, but both species have been reported to shed zoonotic *C. parvum*,^10^ and sheep have been documented with *C. parvum* in India.^11^ The high frequency and shedding of *Giardia* in dogs suggests they may be an important reservoir for *Giardia* and a potentially under-recognized public health concern in the region. In India, semi-domestic and stray dogs are abundant and contaminate surrounding water and soil with fecal material known to harbor a variety of zoonotic pathogens.^12^ Although zoonotic transmission of *Giardia* between humans and dogs is still an open question,^13^ the degree of dog–human interaction in India may increase this risk. The zoonotic potential of protozoa from sheep and dogs should be further investigated in the region.

To examine the contribution of different animal hosts to *Cryptosporidium* and *Giardia* contamination in the study environment, and the potential for environmental exposure to oocysts and cysts shed by animal hosts, we estimated per animal loading rates and total population load by combining information on the per gram geometric mean parasite shedding rates of host animals from this study with published animal fecal production rates^8^,^14^ and 2007 census data on the population of host animals in Puri District.^9^ Geometric mean parasite shedding rate was calculated from the number of parasites per slide well divided by the grams of fecal material used to prepare the slide, including both positive and negative samples. Results are shown in Table 2. Cattle, which represented 61% of Puri District's animal population,^9^ appear to contribute the greatest environmental mass load of *Cryptosporidium* oocysts (2.1E+10/day) and *Giardia* cysts (6.2E+10/day). Previous studies in neighboring West Bengal documented cattle to carry zoonotic strains of *Cryptosporidium* and *Giardia* and noted evidence of zoonotic transmission between cattle and humans,^15^,^16^ raising concern that cattle in Puri District may also harbor genotypes capable of infecting humans.

We also tested village water sources used for drinking (protected groundwater) and domestic hygiene (surface water ponds) for contamination during the 2012 (June–July) and 2013 (June–August) monsoon seasons. Intervention villages (*N* = 50) of the large sanitation trial^7^ were paired with their geographically nearest control village (*N* = 50), and 30 pairs were randomly selected for sampling (60 villages total). In each village, a single 20 L sample was collected from each tested source, and up to six unique sources per village were sampled, comprising two public ponds and two public and private tube wells each (when existent). Samples were transported to the AIPH laboratory on ice and concentrated via ultrafiltration within 8 hours of collection.^17^ A 50 mL aliquot of the retentate (approximately 200 mL) was stored at 4°C for up to 1 week until analyzed at KIIT. IMS-DFA was used for oocyst/cyst enumeration as described previously.^8^

Table 3 shows detection frequency and concentration results by water source type. Ponds were more likely to be contaminated with *Cryptosporidium* (95% CI: 2.9–10.1) and *Giardia* (95% CI: 9.9–33.7) parasites than public or private tube wells (adjusted for intervention status). *Giardia* was detected more frequently (74%) than *Cryptosporidium* (37%) in ponds, consistent with the relative difference in their total environmental loading rates (Table 2). Our finding that *Giardia* was present in 74% of ponds (95% CI: 64–83%) aligns with the GEMS hypothesis of developed immunity because of early and repeated exposure to *Giardia* as an explanation for the higher prevalence they found of *Giardia* among controls than cases, and suggests a larger role for *Giardia* in chronic diarrhea as opposed to acute diarrhea in this and similar settings.^3^ Public (deeper) and private (shallow) tube wells used for drinking had similar, but still unsafe rates of *Cryptosporidium* (10%) and *Giardia* (14%) contamination and oocyst/cyst concentrations. Future work is needed to identify sources and pathways of protozoal contamination of surface and groundwater to reduce exposures to waterborne protozoal infection in study villages.

A subset of fecal and water samples with *Cryptosporidium* and *Giardia* detected at concentrations greater than 10 parasites per DFA slide well were selected for molecular characterization. We choose a threshold of 10 parasites visualized per DFA slide well based on past experience of poor success producing clean sequence data from amplifying environmental samples with fewer visualized parasites, while extracting DNA from samples with greater than 100 parasites visualized is often successful. Nine water samples (1 > 100 parasites), six human (3 > 100 parasites), three pooled dog (0 > 100 parasites), and five pooled livestock (1 sheep, 1 goat > 100 parasites) samples were screened for *Cryptosporidium* and 21 water (3 > 100 parasites), three human (1 > 100 parasites), eight pooled dog (6 > 100 parasites), and eight pooled livestock (3 sheep, 1 cattle > 100 parasites) samples were screened for *Giardia*. For DNA extraction, the sample was subjected to two rounds of freeze boil (4 minutes in liquid N_2_, following immediately by 4 minutes in boiling water) and incubated overnight at 56°C. The remaining extraction steps were carried out per manufacturer instructions (DNeasy Blood and Tissue Kit; Qiagen Inc., Valencia, CA). *Cryptosporidium* isolates were characterized using deoxyribonucleic acid (DNA) sequence analysis of an 18S ribosomal region with primers from Morgan and others.^18^ *Giardia* isolates were characterized using a semi-nested polymerase chain reaction (PCR) and DNA sequence analysis of the glutamate dehydrogenase gene.^19^ Clean *Giardia* DNA sequences were obtained for three pooled dog fecal samples and one human sample. *Giardia* isolates from dogs were identified as host-specific assemblages D (2/3) and C (1/3). One human sample isolate was identified as Assemblage A2. We were able to successfully characterize one *Cryptosporidium*-positive pond water sample as *C. hominis* and five *Giardia*-positive water samples as assemblages A2 (2/5), B (2/5), and C (1/5). Sequences were deposited in GenBank under accession numbers from KJ499989 to KJ499992 and from KR698083 to KR698088.

These findings provide preliminary evidence of the diversity of potential transmission pathways of protozoal diarrheal pathogens and provide an improved understanding of the distribution of *Cryptosporidium* and *Giardia* in coexisting humans and animals, and their shared water sources in a rural region of India. To better explore the role for zoonotic transmission in the region, information on animal ownership and contact would be valuable as well as pairing human and animal fecal samples collected from the same household. Larger and longitudinal cohort studies involving repeated sampling of infants, children, adults, and animals would provide improved estimates of protozoal prevalence as our single sample is likely an underestimate as shown by Kang and others,^20^ who found the sensitivity of detecting gut protozoal pathogens from a single sample to be less than 40%, while with three repeated samples the sensitivity was increased to 80%. Finally, epidemiological studies comparing risks from exposure to different types of contaminated village water sources paired with more extensive molecular characterization of pathogen isolates would provide additional information to practitioners working to reduce the disease burden of fecal protozoa pathogens in India and similar settings.

## Figures and Tables

**Table 1:** Detection of *Cryptosporidium* oocysts and *Giardia* cysts in individual human and pooled domestic animal fecal samples from Odisha, India

Fecal sample type	*n*	*Cryptosporidium*		*Giardia*	
		% Fecal samples positive (95% CI)*	Geometric mean oocysts/10 g feces among positive samples† (range)‡	% Fecal samples positive (95% CI)*	Geometric mean cysts/10 g feces among positive samples† (range)‡
Human	85	12 (7–21)	319 (10–4,909)	12 (7–21)	26 (1–640)
Pooled cattle (5 individuals/pool)	20	5 (0–27)	90 (NA)	40 (20–64)	227 (20–132,810)
Pooled buffalo (4–5 individuals/pool)	22	5 (0–25)	20 (NA)	9 (0–31)	122 (50–300)
Pooled sheep (5 individuals/pool)	20	35 (16–60)	460 (32–181,828)	45 (24–68)	1,118 (20–44,470)
Pooled goat (5 individuals/pool)	20	35 (16–60)	163 (28–2,880)	15 (4–39)	47 (20–170)
Pooled chicken (9–10 individuals/pool)	10	0	NA	0	NA
Pooled dog (4–5 individuals/pool)	18	17 (0–42)	1,236 (361–2,310)	67 (41–86)	4,015 (30–298,880)
Pooled cat (2 individuals/pool)	1	0	NA	100 (NA)	119,680 (NA)

CI = confidence interval; NA = non-applicable.

* Binomial CIs.

† Positive samples are those for which parasites were visualized on slides as indicated in the adjacent column to the left.

‡ Geometric mean count and range is from subset of samples positive for protozoa detected by Immunomagnetic separation and direct immunofluorescent antibody tests (IMS-DFA).

**Table 2:** Environmental loading of *Cryptosporidium* oocyst and *Giardia* cyst from five domestic animal host groups in Puri District, Odisha, India

Host species	Puri District 2007 population (*N*)	Feces production rate* g/day/individual	*Cryptosporidium*		*Giardia*	
			Loading rate† oocyst/day/individual (±2σ)‡	Total load for Puri District oocyst/day (±2σ)‡	Loading rate† cyst/day/individual (±2σ)‡	Total load for Puri District cyst/day (±2σ)‡
Cattle	429,397	38,400	4.8E+04 (1.8E+04, 1.3E+05)	2.1E+10 (7.7E+09, 5.6E+10)	1.5E+05 (1.2E+03, 1.7E+07)	6.2E+10 (5.3E+08, 7.1E+12)
Buffalo	27,401	38,400	3.6E+04 (1.8E+04, 7.0E+04)	9.8E+08 (4.9E+08, 1.9E+09)	4.3E+04 (7.5E+03, 2.4E+05)	1.2E+09 (2.0E+08, 6.6E+09)
Goat	132,717	1,664	6.4E+03 (3.4E+02, 1.2E+05)	8.5E+08 (4.5E+07, 1.6E+10)	3.4E+03 (1.1E+03, 1.0E+04)	4.5E+08 (1.5E+08, 1.4E+09)
Sheep	67,466	675	3.4E+03 (2.7E+01, 4.3E+05)	2.3E+08 (1.8E+06, 2.9E+10)	7.2E+03 (3.2E+01, 1.6E+06)	4.9E+08 (2.2E+06, 1.1E+11)
Dog	45,347	249	6.7E+02 (1.7E+01, 2.6E+04)	3.0E+07 (7.8E+05, 1.2E+09)	1.4E+04 (5.3E+00, 4.0E+07)	6.6E+08 (2.4E+05, 1.8E+12)
Total	702,328	–	–	2.3E+10 (8.2E+09, 1.0E+11)	–	6.5E+10 (8.9E+08, 9.2E+12)

Estimated from the 2007 animal population census and shedding rates measured in this study.

* Feces production/day (gram) based on published literature for cattle, sheep, and goat,^14^ and for dog.^8^ No published information could be found for buffalo, so the cattle fecal production rate has been used.

† Loading rate per individual for each host species was calculated as the product of the overall geometric mean shedding rate found in this study (positive and negative samples) and the fecal production rate as indicated. Negative samples were assigned half the sample lower detection limit for geometric mean calculations.

‡ Two standard deviation (±2σ) from the geometric mean of loading rates provided to indicate level of loading rate variability.

**Table 3:** Detection of *Cryptosporidium* oocysts and *Giardia* cysts in 301 village drinking and domestic water sources sampled in 60 villages in Puri District, Odisha, India

Village water sources	*n*	*Cryptosporidium*		*Giardia*	
		% Water samples positive (95% CI)*	Geometric mean oocysts/20 L of water among positive samples† (range)‡	% Water samples positive (95% CI)*	Geometric mean cysts/20 L of water among positive samples† (range)‡
Public ponds (surface water)	94	37 (27–48)	84 (10–5,968)	74 (64–83)	137 (10–69,640)
Public tube wells (deep groundwater)	111	14 (8–21)	22 (8–110)	12 (7–19)	28 (9–520)
Private tube wells (shallow groundwater)	96	5 (2–12)	19 (8–115)	17 (10–26)	25 (9–70)
Public and private tube wells combined (drinking water)	207	10 (6–15)	21 (8–115)	14 (10–20)	26 (9–520)

CI = confidence interval.

* Binomial CIs.

† Positive samples are those for which parasites were visualized on slides as indicated in the adjacent column to the left.

‡ Geometric mean count and range is from subset of samples positive for protozoa detected by Immunomagnetic separation and direct immunofluorescent antibody tests (IMS-DFA).
